# Hours Worked and Clinical Workforce Equivalent Patterns in the Pediatric Subspecialty Workforce

**DOI:** 10.1001/jamanetworkopen.2025.2956

**Published:** 2025-03-31

**Authors:** Colin J. Orr, Laurel K. Leslie, Andy Knapton, Adam L. Turner, Erin P. Fraher

**Affiliations:** 1 Department of Pediatrics, Vanderbilt University Medical Center, Nashville, Tennessee; 2 American Board of Pediatrics, Chapel Hill, North Carolina; 3 Tufts University School of Medicine, Boston, Massachusetts; 4 Strategic Modelling and Analysis Ltd, Winchester, United Kingdom; 5 Cecil G. Sheps Center for Health Services Research, University of North Carolina at Chapel Hill, Chapel Hill; 6 Department of Family Medicine, University of North Carolina at Chapel Hill, Chapel Hill

## Abstract

This cross-sectional study examines the self-reported allocation of work over a 10-year period by US pediatricians in 14 subspecialties.

## Introduction

There are questions about the pediatric subspecialty workforce’s ability to meet the increasing health needs of children.^1,2^ We examined patterns in pediatric subspecialists’ self-reported hours worked and time spent in clinical care.

## Methods

This repeated cross-sectional analysis was conducted between June and September 2024 using data from the American Board of Pediatrics (ABP) Maintenance of Certification (MOC) surveys from 2009 to 2019 for the 14 ABP-certified subspecialties. Pediatric hospital medicine was excluded because it was first offered in 2019. The University of North Carolina at Chapel Hill Institutional Review Board approved the study and waived informed consent because an existing dataset was analyzed. We followed the STROBE reporting guideline.

MOC surveys ask pediatricians to self-report the mean number of hours worked per week and the proportion of time spent on direct and/or consultative inpatient and outpatient care, including patient billing and medical record review and documentation (with or without trainees), over the past 6 months.^3^ Two outcomes were derived: hours worked per week and clinical workforce equivalent (CWE; calculated by multiplying mean weekly hours worked by proportion of time in clinical care). Descriptive and linear regression analyses were conducted. Exposures of interest were MOC survey year and year measured by indicator variables. Covariates included age, subspecialty, and degree type (US: doctor of medicine, doctor of osteopathic medicine; International Medical Graduate [IMG]). Huber-White SEs accounted for clustering at the individual level. Data analysis was performed with Stata 17 (StataCorp).

## Results

The sample included 26 776 observations representing 17 052 unique pediatric subspecialists (8697 males [51%], 8355 females [49%]; 9208 [54%] aged <50 years). Most subspecialists (84.7%) had medical degrees and graduated from US medical schools. In unadjusted models, between 2009 and 2019, CWE was lower than hours worked per week (Table 1). Females reported working fewer hours and a lower CWE compared with males during the study (Table 1). However, from 2009 to 2019, the decrease in hours worked was greater in males than females (9.1 vs 6.2 fewer hours).

**Table 1.** Unadjusted Self-Reported Hours Worked and CWE Among Pediatric Subspecialists (N = 26 776 Survey Responses)^a^

MOC survey year	Unadjusted self-reported hours worked per week, Mean (SD)			Unadjusted CWE (SD)^b^		
	Female	Male	Absolute difference by gender	Female	Male	Difference by gender
2009	56.5 (16.3)	62.4 (13.7)	5.9	36.2 (17.3)	39.8 (17.3)	3.6
2010	56.7 (16.2)	61.3 (14.7)	4.6	35.8 (17.2)	38.9 (17.4)	3.0
2011	56.1 (15.8)	61.3 (14.3)	5.2	36.7 (16.7)	39.2 (17.8)	2.5
2012	55.55 (15.3)	60.9 (13.6)	5.5	36.8 (16.6)	39.5 (17.7)	2.7
2013	53.3 (14.3)	61.8 (14.2)	8.5	36.0 (18.0)	43.4 (15.7)	7.4
2015	50.9 (12.8)	54.6 (10.3)	3.7	33.2 (15.0)	35.5 (15.4)	2.3
2016	49.4 (12.8)	54.3 (10.4)	4.9	33.1 (13.9)	35.2 (14.7)	2.1
2017	50.7 (11.7)	54.4 (10.1)	3.7	34.1 (13.7)	36.0 (14.2)	2.0
2018	49.1 (11.9)	51.9 (10.7)	2.7	32.6 (14.3)	32.8 (14.3)	0.3
2019	50.5 (12.1)	53.3 (10.9)	2.9	32.2 (14.5)	33.6 (15.3)	1.4
Difference 2009-2019	−6.00	−9.1	3.0	−4.0	−6.2	2.2

Abbreviations: CWE, clinical workforce equivalent; MOC, Maintenance of Certification.

^a^ Included are 26 776 responses from 17 052 survey participants. Duplicate responses were allowed as the survey was offered every 5 years. Data from the 2014 MOC survey were not available due to a data processing issue.

^b^ CWE was calculated by multiplying self-reported mean weekly hours worked by proportion of time spent on direct and/or consultative inpatient and outpatient care, including patient billing and medical record review and documentation (with or without trainees).

In adjusted models, subspecialists reported working 7.7 (95% CI, −8.4 to −7.0) fewer hours per week and a 5.3 (95% CI, −6.1 to −4.5) lower CWE in 2019 vs 2009 (Table 2). Females worked a mean of 3.4 (95% CI, −3.8 to −3.0) fewer hours than males and had a 1.2 (95% CI, −1.7 to −0.7) lower CWE than males. Compared with neonatology, there were significant decreases in hours worked in 7 subspecialties and decreases in CWE in all subspecialties except cardiology (Table 2). IMG degree, age (≥60 years), and MOC survey years (2012 and 2015-2019) were associated with decreases in total hours worked. Age (40-44 and ≥60 years) and MOC survey years (2015-2019) were associated with decreases in CWE.

**Table 2.** Adjusted Self-Reported Total Hours Worked and CWE (N = 17 052 Pediatric Subspecialists)^a^

Characteristics	Mean (95% CI)	
	Self-reported total hours worked	CWE
Subspecialty		
Neonatology	1 [Reference]	1 [Reference]
Adolescent	−5.5 (−6.8 to −4.3)	−9.6 (−11.1 to −8.2)
Cardiology	0.6 (−0.1 to 1.3)	2.3 (1.5 to 3.1)
Child abuse	−5.0 (−6.8 to −3.3)	−8.0 (−9.7 to −6.2)
Critical care	1.8 (1.1 to 2.4)	−3.2 (−4.1 to −2.4)
Developmental and behavioral	−6.3 (−7.4 to −5.2)	−5.5 (−6.8 to −4.3)
Emergency medicine	−11.7 (−12.4 to −10.9)	−10.2 (−10.9 to −9.5)
Endocrinology	−3.8 (−4.6 to −3.0)	−4.8 (−5.9 to −3.8)
Gastroenterology	−1.0 (−1.8 to −0.3)	−1.2 (−2.2 to −0.2)
Hematology-oncology	1.0 (0.3 to 1.7)	−8.2 (−9.1 to −7.3)
Infectious disease	−2.2 (−3.1 to −1.4)	−14.3 (−15.5 to −13.1)
Nephrology	−0.1 (−1.2 to 1.0)	−4.5 (−6.1 to −3.2)
Pulmonology	−0.7 (−1.5 to 1.0)	−3.4 (−4.5 to −2.2)
Rheumatology	−1.3 (−2.7 to 0.1)	−7.5 (−9.4 to −5.5)
Gender		
Male	1 [Reference]	1 [Reference]
Female	−3.4 (−3.8 to −3.0)	−1.2 (−1.7 to −0.7)
Degree type		
US: MD	1 [Reference]	1 [Reference]
US: DO	0.2 (−0.8 to 1.1)	3.6 (2.4 to 4.8)
IMG^b^	−0.7 (−1.2 to −0.1)	1.8 (1.1 to 2.4)
Age, y		
<40	1 [Reference]	1 [Reference]
40-44	0.7 (0.3 to 1.2)	−0.7 (−1.3 to −0.1)
45-49	1.4 (0.8 to 1.9)	−0.5 (−1.1 to 0.2)
50-59	1.8 (1.3 to 2.4)	−0.1 (−0.8 to 0.6)
55-59	1.8 (1.3 to 2.4)	−0.3 (−1.0 to 0.4)
≥60	−0.6 (−1.2 to 0.5)	−1.9 (−2.7 to −1.1)
MOC survey year		
2009	1 [Reference]	1 [Reference]
2010	−0.4 (−1.2 to 0.4)	−0.4 (−1.4 to 0.6)
2011	−0.7 (−1.6 to 0.13)	0.9 (−0.1 to 1.9)
2012	−1.6 (−2.5 to −0.8)	0.0 (−1.0 to 1.0)
2013	−1.6 (−4.3 to 1.0)	1.9 (−1.1 to 5.0)
2015	−7.0 (−7.7 to −6.4)	−3.5 (−4.3 to −2.7)
2016	−7.1 (−7.8 to −6.4)	−2.7 (−3.6 to −1.9)
2017	−7.4 (−8.1 to −6.7)	−3.5 (−4.3 to −2.6)
2018	−7.9 (−8.7 to −7.1)	−4.1 (−5.0 to −3.2)
2019	−7.7 (−8.4 to −7.0)	−5.3 (−6.1 to −4.5)

Abbreviations: CWE, clinical workforce equivalent; DO, doctor of osteopathic medicine; IMG, International Medical Graduate; MD, doctor of medicine; MOC, Maintenance of Certification.

^a^ Linear regression models adjusted for age, gender, subspecialty, degree type (MD, DO, IMG), and MOC survey year. Huber-White SEs were used to account for clustering at the individual level. Data from the 2014 MOC survey year were not available due to a data processing issue.

^b^ IMG includes MD and medical degrees from outside the US.

## Discussion

We found decreases in hours worked and CWE from 2009 to 2019. Females reported working fewer hours weekly and had lower CWEs than males. Overall decrease in CWE is surprising given the increasing acuity of children’s health needs.^4^ The decline in hours and CWE may impede access to pediatric subspecialty care. More research is needed to understand the factors underlying this decrease and its implications for access and outcomes. The findings suggest that additional measures beyond total hours worked or absolute numbers of subspecialists are necessary when analyzing clinical capacity. Measures beyond CWE could include the ratio of new patient visits to return visits or number of visits before returning to primary care.

Study limitations were self-reported data, which may not reflect actual allocation of effort; different response rates by year, which could introduce nonresponse bias; unknown clinical significance of decreases in hours worked and CWE; and data being from before the COVID-19 pandemic. It will be important to monitor postpandemic patterns.

Practice patterns of the pediatric subspecialty workforce are changing, with variations by year and gender that may have implications for access to pediatric subspecialty care. Future workforce analyses should use measures that more accurately capture clinical effort.

## Notes

Supplement.
Data Sharing Statement
