# Supplementary material for: Hours Worked and Clinical Workforce Equivalent Patterns in the Pediatric Subspecialty Workforce
Source: JAMA Netw Open. 2025 Mar 31;8(3):e252956. doi: 10.1001/jamanetworkopen.2025.2956 (PMC11959437; doi:10.1001/jamanetworkopen.2025.2956)
Supplement: Supplement. — Data Sharing Statement [file jamanetwopen-e252956-s001.pdf]

Data Sharing Statement

Orr. Hours Worked and Clinical Workforce Equivalent Patterns in the Pediatric Subspecialty Workforce. *JAMA Netw Open*. Published March 31, 2025.
doi:10.1001/jamanetworkopen.2025.2956

Data
Data available: No

Additional Information
Explanation for why data not available: Private data of the American Board of Pediatrics. Want to protect the confidentiality of survey respondents.
